# A Cost-Benefit Analysis of Soil Disinfestation Methods against Root-Knot Nematodes in Mediterranean Intensive Horticulture

**DOI:** 10.3390/plants11202774

**Published:** 2022-10-19

**Authors:** Miguel Talavera-Rubia, María Dolores Vela-Delgado, Soledad Verdejo-Lucas

**Affiliations:** 1 Andalusian Institute of Agricultural and Fisheries Research and Training (IFAPA), IFAPA Centro Alameda del Obispo, Av. Menéndez Pidal s/n, 14004 Cordoba, Cordoba, Spain; 2 Andalusian Institute of Agricultural and Fisheries Research and Training (IFAPA), IFAPA Centro Rancho de la Merced, Carretera Cañada de la Loba CA-3102, Km. 3,1, 11471 Jerez de la Frontera, Cadiz, Spain; 3 Andalusian Institute of Agricultural and Fisheries Research and Training (IFAPA), IFAPA Centro La Mojonera, Autovía del Mediterráneo, Salida 420, Paraje San Nicolás, 04745 La Mojonera, Almeria, Spain

**Keywords:** cost-benefit, nematicides, *Meloidogyne incognita*, vegetables

## Abstract

Losses caused by phytoparasitic nematodes in crops depend directly on their soil densities at the start of the crop, so reducing their populations before planting is the main aim of nematological management. Efficacies in reducing *Meloidogyne* soil populations of soil disinfestation methods, such as agrochemicals, botanicals, or biosolarization were estimated on multiple field trials conducted over fourteen years in intensive horticultural crops. Soil nematode populations were reduced by 87 to 78% after fumigation with 1,3-dichloropropene + chloropicrin and dimethyl-disulphide, respectively. Non-fumigant nematicides such as azadirachtin, dazomet, fenamiphos, fluopyram, fosthiazate, metam-sodium, and oxamyl showed efficacies ranging from 51 to 64%, whereas the efficacy of natural products, such as abamectin, garlic extracts, or essential oils was 41 to 48%. Biosolarization with chicken manure had an efficacy of 73%. An economic cost-benefit study of nematode management methods was performed for seven vegetable–*M. incognita* pathosystems. Fumigation with 1,3-dichloropropene + chloropicrin and biosolarization with chicken manure were the only treatments able to reduce RKN populations above 1000 and 750 J2 per 100 cm^3^ of soil, respectively, to levels below the nematode economic damage threshold, keeping profitability. Fumigation was able to manage RKN soil densities up to 350 J2 per 100 cm^3^ of soil in most susceptible crops as aubergine or cucumber and up to 1000 J2 per 100 cm^3^ of soil for more tolerant crops, such as other cucurbits, pepper, or tomato. Other nematicidal treatments were not able to reduce RKN populations above 200–300 J2/100 cm^3^ of soil below the economic thresholds but were profitable when RKN densities were below the limits of 200–300 J2/100 cm^3^ of soil.

## 1. Introduction

Intensive horticulture is a key agricultural industry in southern Europe. High-value crops such as cucurbitaceous (cucumbers, melons, watermelons, and zucchinis) and solanaceous crops (aubergines, peppers, and tomatoes) are grown under plastic protection in unheated greenhouses during the autumn–winter months and are exported for fresh consumption to European markets and elsewhere. In south-eastern Spain, there are over 40,000 ha of protected vegetable crops producing around 4,000,000 tons, with a commercial value of about 3000 million € per year [1].

Plant parasitic nematodes are major limiting factors for vegetable production worldwide [2,3]. Estimated worldwide losses caused by nematodes are about 12% of the total yield, valued at $157 billion annually [3]. Root-knot nematodes (RKN: *Meloidogyne* spp.) are seen as the most impacting and frequent limiting factor to achieving premium quality and economically sustainable yields in intensive horticulture [4,5]. RKN prevalence ranges from 20 to 70% in southern European intensive horticulture and constitutes a key limiting factor to achieving economically sustainable yields [4].

Yield losses caused by nematodes are related to nematode densities at planting (Pi) and the relationship was mathematically described by the Seinhorst damage function model [6]. This model provides indicators of plant tolerance: the tolerance limit (T: Pi below which plant growth or yield is not affected) and the minimum yield (m) at high Pi, as a percentage of the maximum yield that would be obtained in the absence of nematodes. Therefore, Pi has been used as a predictor of nematode damage in crops, and according to Seinhorst damage models, RKN soil densities at planting (Pi) should be ideally below the tolerance limit, so not to cause any appreciable yield losses.

Since nematode-caused yield losses are related to Pi, nematode management methods have been based on reducing the nematode densities before the crop is planted. To keep the profitability of the nematicidal treatments, their cost should at least be balanced with the yield losses that will be avoided. The nematode density at which the value of the yield losses are equal to the cost of management practice is known as the economic threshold (ET) [7], and it can be calculated from the Seinhorst damage model. When pre-treatment nematode densities (P0) are below the ET, application of the nematicidal treatment will not be profitable since its cost will be higher than the yield losses that nematodes cause, but when P0 are higher than the ET, the nematicidal treatment will be profitable if it can reduce the nematode densities to levels at planting (Pi) below the ET. Knowing the nematicidal efficacy of a treatment would allow us to estimate the maximum P0 densities that the treatment could manage to get the Pi at densities below the economic threshold.

Conventionally, the management of RKN has relied on soil disinfestation by chemical nematicides, which are classified as fumigant (gas) and non-fumigant (contact agents). All these nematicides are broad-spectrum pesticides effective in reducing RKN soil populations, but they may have undesirable side effects on beneficial soil organisms [8]. Increasing social concern about the environmental risks involved in the use of chemicals in agriculture has restricted their use in many countries. Currently, seven chemical nematicides (abamectin, dazomet, fosthiazate, fluopyram, metam sodium, metam potassium, oxamyl), two botanical pesticides (based on garlic extracts and essential oils), and two biological control agents (*Bacillus firmus* and *Purpureocillium lilacinum*) are approved for use against RKN in the European Union (EU) [9]. Other agrochemicals such as dimethyl-disulphide and fluazaindolizine are under revision, though EU member states can allow temporary authorizations for emergency uses in particular agricultural industries (Regulation 1107/2009 article 53) [9]. Despite of 1,3-dichloropropene, and chloropicrin are currently “not approved” for use within the EU, fumigation with 1,3-dichloropropen + chloropicrin has been considered the most effective method to manage RKN disease in intensive horticultural crops by farmers in southern Europe, since other methods have not provided enough consistency when high RKN soil populations occur [4,5,10]. Newly developed nematicides, such as fluensulfone or tioxazafen, are not registered for use nor waiting for approval in the EU [11]. Physical and cultural methods, such as plant resistance, biofumigation, solarization, and biosolarization are also used, but they are limited to the availability of resistant genotypes or sufficient organic amendment supply [12]. Therefore, if soil fumigants are eventually banned, other strategies based on the integration of several management methods will be necessary to manage RKN diseases, providing that the economic viability of the crop is maintained.

The economic return of a crop is determined by its yield, costs associated with production, and the price obtained. Cost estimations assume a static price for land rent and average prices for input costs and management practices. This information is regularly available to growers through periodic publications by agricultural extension services of the regional government [13]. However, they do not usually include the cost of nematicides that contribute to the total production cost and may reduce economic returns. The cost of reducing RKN populations depends on the price of the nematicidal products, the labour associated with their application, and their efficacy. The profitability of any RKN management method can be modelled when relationships between RKN soil densities at planting (Pi) and crop yield, nematicide cost and efficacy, and crop value and net returns are known, and it will be maximized when the difference between the revenue obtained from the crop and the cost of nematode management is greatest [7,14]. Costs associated with nematode management have been examined previously in cotton [15,16,17] and potato [18], but no assessments have been made on intensive horticultural crops, which currently are the most demanding systems for nematicidal products [4,5,10].

Integrated nematode management systems (INM) should be tailored for each crop-nematode pathosystem based on the scientific knowledge on the plant-nematode interaction, nematicidal efficacy, costs and profit balance, and environmental and health side effects of the management methods. However, due to the limited availability of data, a great deal of uncertainty exists as to how growers will deal with high RKN infestations in intensive horticulture. Nematicide performance models, including effectiveness, financial and environmental aspects, are therefore crucial tools for the design of INM and should be adopted to advise growers through agricultural extension activities [14].

This paper examines nematicidal efficacies, economic cost, net returns, and profitability of current RKN management methods in the European Union based on field data and statistical sources for the main vegetable crops in intensive horticulture. The main objective of this study aims at generating information to help decision-making for nematode management decision making, according to the various attributes involved, such as nematode densities previous to any nematicidal treatment and at planting, the cost of the nematicidal treatments, their field efficacy, and their economic threshold.

## 2. Results

The most efficient treatments against RKN were soil fumigation with 1,3-dichloropropene + chloropicrin and dimethyl disulphide, which reduced RKN densities by 87% and 78%, respectively (Table 1). Metam-sodium showed a lower efficacy in reducing RKN densities (51%). Non-fumigant nematicides, such as fluopyram, oxamyl, fluazaindolizine, dazomet, fosthiazate, and fenamiphos, showed medium efficacies ranging from 51 to 64%. The efficacies of biological pesticides or essential oils and garlic extract were less than 50%. Biosolarization with chicken manure reduced RKN densities by 78%, it was less effective than 1,3-dichloropropene + chloropicrin, but equivalent to the efficacy of dimethyl disulphide and superior to most non-fumigant nematicides.

The cost of the nematicidal treatments ranged from 160 €/ha for a single oxamyl treatment to 2700 €/ha for biosolarization with chicken manure (Table 1).

Production costs, yields, revenues, and net returns for vegetable intensive cultivation in south Spain are summarized in Table 2.

Tolerance limits (T) and minimum yields at the highest nematode densities (m) were calculated from the Seinhorst damage function models reported in the literature (Table 3).

For solanaceous crops, economic thresholds varied from 10 to 87 J2/100 cm^3^ of soil for aubergine, 51–218 for pepper, and 98–205 for tomato (Table 4). The net return losses caused by RKN at these economic thresholds varied between 0.6% and 11.3% of the total net return that would be obtained in the absence of nematodes (Table 4).

For cucurbitaceous crops, nematode economic thresholds varied from 6 to 346 J2/100 cm^3^ of soil. The net return losses caused by RKN at these economic thresholds varied between 0.8% and 27.1% of the total net return that would be obtained in the absence of nematodes in cucurbitaceous crops (Table 5).

Maximum RKN soil densities (P0) that each nematicidal treatment could manage to get RKN soil densities at planting (Pi) at the economic threshold level were calculated according to the nematicidal efficacies obtained in field trials (Table 6 and Table 7).

## 3. Discussion

Negative net returns were obtained at the hypothetical highest RKN densities for all vegetable–RKN pathosystems, which proves the necessity for nematode management in these horticultural intensive crops, in case of high RKN soil infestations. Pi levels higher than 200 J2/100 cm^3^ of soil before planting are found in some fields dedicated to intensive horticulture in southern Spain [4].

Nematicidal efficacies, expressed as the reduction in RKN populations due to the nematicidal treatment after removing the natural RKN mortality in soil showed and ample range of variation from 40% to 87% (Table 1).

The superior efficacy of soil chemical fumigation against RKN has been previously reported in vegetable crops [4,9,10,11]. Greco et al. reported that in RKN highly-infested soils, chemical fumigants reduced root crop infestation by 71–74% and increased crop yields by 289–336% [10]. These results agree with the agricultural advisors’ opinion on the efficacy of nematicide treatments since they also consider fumigant nematicides as the most effective method against RKN in vegetable crops [4]. The efficacies of the biological products *B. firmus* or *P. lilacinus* could not be determined since their modes of action do not reduce RKN soil densities but protect the plant from nematode infection or parasitize the RKN eggs.

The most tolerant crops to *M*. *incognita* were tomato and pepper, with average tolerance limits of about 92 and 41 J2/100 cm^3^ of soil, respectively. These tolerance limits were averaged from the values reported in the literature in pots and field experiments and depend on the local conditions in which the experiments were carried out, soil type, and temperature, crop cultivar, and the RKN inoculum used, showing in some cases a high variability. Tomato tolerance limits to *M*. *incognita* varied between 2 and 400 J2/100 cm^3^ of soil [26,27,28,29,30], and pepper tolerance limits between 8 and 74 J2/100 cm^3^ of soil [24,25]. Aubergine and cucurbits were less tolerant to the infection by *M*. *incognita* with tolerance limits below 6 J2/100 cm^3^ of soil [19,20,21,22,23,31,32], being cucumber the most susceptible crop with a tolerance limit of 0.1 J2/100 cm^3^ of soil [20]. The crop that can suffer the highest yield losses caused by *M*. *incognita* was aubergine (95%), but at the highest RKN densities, pepper and tomato can lose up to 52% and 69% of the yield, respectively. Cucurbit maximum yield losses varied between 57 and 80%.

The nematode economic threshold varied between 6 and 346 J2/100 cm^3^ of soil, depending on the susceptibility-tolerance of the crop to the RKN species, the yield revenue, and the cost of the nematicidal treatment. When nematode densities were above the ET, the nematicidal treatment would be economically justified since the increases in the net return obtained would be higher than the cost of the nematicide treatment. These nematode economic damage thresholds assumed yield losses from 0.6 to 27.1% of the net return, a highly variable range, depending on the susceptibility-tolerance of the crop to the RKN species, the yield revenue, and the cost of the nematicidal treatment. Most nematicidal treatments were under the cost of 1000 €/ha and showed economic thresholds from 6 to 110 J2/100 cm^3^ of soil, with associated net return losses 1–10%. More expensive treatments (1550–2700 €/ha) had economic thresholds from 51 to 306 J2/100 cm^3^ of soil and associated net return losses from 5% up to 27%. The assumption of these net return losses by farmers will depend on the total revenue obtained, being more acceptable in high-valued crops since the higher revenues obtained will compensate for higher nematicidal costs. All costs for non-fumigant nematicides in the study were based on one single application before planting, but some of them can be additionally applied several times in post-transplanting, when the crop is growing. In such cases, the costs would be increased, but also the efficacies in controlling RKN diseases and the net return obtained. These variations in the nematicide application should be considered when assessing the profitability of a nematicidal treatment if more than one application of the product is to be done.

The economic thresholds do not consider nematicidal efficacies in their calculation, but it is a critical issue to assess the economic profitability of treatment because more efficient treatments can reach the economic threshold from higher field RKN densities than less efficient treatments. Thus, the incorporation of nematicidal efficacies into nematicide performance models will allow us to estimate a range of field P0 that each treatment could afford to keep profitability. For example, such a P0 range for 1,3-dichloropropene + chloropicrin (87% efficacy) was 51–398 *M*. *incognita* for aubergine, 58–453 for cucumber, 137–1070 for pepper, 154–1203 for zucchini, 155–1211 for tomato, 160–1250 for watermelon, 181–1414 for melon (Table 4, Table 5, Table 6 and Table 7). Even for the most susceptible crops, as aubergine and cucumber soil fumigation with 1,3-dichloropropene + chloropicrin could reduce high *M*. *incognita* soil densities, over 400 J2/100 cm^3^ of soil to the economic threshold levels at planting, keeping yield and net return losses lower than the cost of the nematicidal treatment. In less susceptible crops, fumigation with 1,3-dichloropropene + chloropicrin could reduce RKN populations over 1000 J2/100 cm^3^ of soil to economic threshold levels keeping profitability. The widespread use of fumigant nematicides in intensive horticulture can be explained according to these results, from their efficacies in reducing RKN soil densities, since even with one of the highest costs (1550 €/ha), they still result in the treatments that can manage the highest RKN soil densities keeping profitability.

The most expensive treatment, biosolarization with chicken manure (2700 €/ha) and a 72–73% efficacy was profitable within a P0 range of 87–322 *M. incognita* J2/100 cm^3^ of soil for aubergine, 103–381 for cucumber, 205–759 for tomato, 218–807 for pepper, 293–1085 for zucchini, 306–1133 for watermelon, and 346–1281 for melon. Biosolarization with chicken manure at the cost of 2700 €/ha, could still be profitable for the most susceptible crops, aubergine, and cucumber, providing RKN soil densities in field plots that were under 322–388 J2/100 cm^3^ of soil. In less susceptible crops, profitability was kept even at high RKN soil infestation (>750 J2/100 cm^3^ of soil).

For less efficient nematicidal treatments (50–65%) with a cost below 1000 €/ha (160–970 €/ha), the P0 ranges in which the nematicidal treatment would be profitable were 6–81 *M. incognita* J2/100 cm^3^ of soil for cucumber, 11–76 for aubergine, 16–209 in zucchini, 16–215 in watermelon, 22–247 in melon, 51–224 for pepper, and 98–294 in tomato. Therefore, the use of these nematicidal treatments would not be profitable in those field plots where RKN soil densities were above 76–81 J2/100 cm^3^ of soil if the crop to be planted were aubergine or cucumber and profitability of the treatment would also be compromised in less susceptible crops where RKN soil densities were above 300 J2/100 cm^3^ of soil.

Overall, when the P0 are within these ranges, the Pi after the nematicidal treatment result in net return losses lower than the treatment costs, and therefore, treatments will be clearly profitable. In case of P0 densities below the lower limits of these ranges (Table 4 and Table 5: ET columns), the treatment costs are higher than the net return losses caused by nematodes, and the application will not be worth it economically. P0 densities above the upper limits (Table 6 and Table 7) of these ranges result in Pi densities at planting higher than the economic threshold, and thus, net return losses will be higher than treatment costs. In general, when the costs of the nematicidal treatment increase, the economic threshold also rises, and when the nematicidal efficacies are lower, the maximum P0 that the treatments can afford to maintain profitability decreases, narrowing the ranges of profitability. For instance, When P0 populations were above 200–300 J2/100 cm^3^ of soil, some nematicidal treatments would not be able to reduce RKN densities below the economic thresholds in most susceptible crops, such as aubergine or cucumber, keeping profitability. In such cases, additional RKN management methods should be applied to further reduce RKN densities from P0 to Pi, or to reduce the yield losses caused by nematodes, i.e., the use of resistant cultivars or the use of biocontrol agents that can reduce multiplication of nematodes once the roots are infested.

## 4. Materials and Methods

### 4.1. Assessment of Nematicidal Efficacies

Data on the relative efficacy of various nematicidal treatments in reducing RKN soil populations were obtained from a series of field trials carried out during the period 2007–2021 in the Andalusian Institute of Agricultural and Fisheries Research and Training (IFAPA) at a plastic greenhouse located at the IFAPA Chipiona experimental station, Cádiz, Spain (36°45′ N—6°24′ W) used for regular cultivation of vegetables and naturally infested with *Meloidogyne incognita* (loamy sand soil, pH 7.3, electric conductivity 0.75 mS/cm, organic matter 1.6%).

In each field trial, up to twelve different nematicidal treatments plus an untreated control were set in a randomized complete block design and distributed over thirty-nine plots (30 m^2^). Each single nematicidal treatment was replicated three times and included in four different field trials (*n* = 12).

Before any treatment, the soil of each individual plot was completely tilled and mixed by crosswise ploughing and subsequently irrigated with a sprinkler for two consecutive days to moisten the soil to a depth of 30 cm. Chemical nematicides were applied by drip irrigation under a low-density polyethylene film (0.03 mm thick) in cultivation lines, except for granular products that were distributed and mixed with the soil of the cultivation lines and watered by drip irrigation according to manufacturer instructions. All chemical treatments were applied at the doses indicated by the manufacturer 4–6 weeks before planting (Table 8). Biosolarization was done each season in mid-July. Chicken manure from nearby chicken farms was evenly distributed over the soil surface and then incorporated into the 20 cm top layer by transverse ploughing using a cultivator. Plots were then drip-irrigated until the soil reached field capacity and covered with a low-density transparent polyethylene film (0.03 mm thick) for about 6 weeks. The polyethylene films were then removed, and the soils were prepared for planting.

To determine the nematicidal efficacy of each treatment, changes in RKN soil densities were recorded two times; pre-treatment (P0) and pre-planting (Pi). At each sampling time, ten cylindrical soil cores were taken per plot using an Auger sampling tool (2 cm in diameter to 30 cm deep) and the soil cores were mixed into a single composite soil sample. Nematodes were extracted from subsamples of 250 cm^3^ of the mixed soil by the Whitehead and Hemming tray method [33]. Only plots with P0 higher than 50 RKN juveniles (J2) per 100 cm^3^ of soil were included in the respective trials. The relative nematicidal efficacies were determined using the Schneider–Orelli correction [34], based on reductions in soil nematode densities from P0 to Pi and corrected for natural mortality in the untreated control plots of the corresponding trial. Efficacies were calculated for each field trial separately due to seasonal and site variations.

mortality = [1 − (Pi/P0)](1)

Schneider–Orelli corrected efficacy = [(mt − mc)/(1 − mc)] × 100(2)

where “m” is the mortality rate in a treated sample, and “mc” is the mortality rate in the untreated control.

### 4.2. Cost-Benefit Analysis

A cost-benefit analysis was conducted to assess the economic profitability of each nematicidal treatment. Cost and profits were estimated for each crop based on agricultural statistical data for the 2019–2021 seasons [13]. The net returns for each crop were calculated by subtracting the production costs from the total revenue (average yield × average price).

Yield losses caused by RKN were estimated according to the Seinhorst damage function models, which relate yield losses to the nematode soil densities at planting time (Pi) [35].

Y = m + (1 − m)z^(Pi−T)^ (3)

where T = tolerance limit (nematode density below which there is no yield loss), m = the minimum yield (obtained at maximum nematode densities), Pi = nematode densities at planting, z = a constant ≤1 and Y = the relative yield, expressed as the rate of the total yield obtained in the absence of nematodes.

Nematicide prices were quoted from local vendors. From February to November 2020, a poll on the nematicide costs was carried out by face-to-face interviews with nine local vendors in intensive horticultural areas of south Spain. An average cost for each nematicidal treatment was calculated for a single application of the product before planting, according to the maximum dosage recommended by manufacturers for intensive horticultural crops. Nematode economic thresholds (ET), defined as the population density at which the value of the yield loss equals the cost of the management method, were calculated for each nematicidal treatment using the Seinhorst damage function models, with the relative yield (Y) expressed as net return values in €/ha [7]. Maximum P0 densities that a nematicidal treatment could reduce to get Pi levels equal to the economic threshold were calculated according to the nematicidal efficacies obtained in the field trials.

## 5. Conclusions

At current nematicide prices, most nematicidal treatments were able to manage RKN soil infestations of about 200 J2/100 cm^3^ of soil, keeping profitability for all vegetable crops in intensive horticulture in South Spain. The only nematicidal treatments that could manage high RKN soil infestation, above 350–400 *M*. *incognita* J2/100 cm^3^ of soil, in all intensive horticultural crops while keeping profitability were 1,3-dichloropropene + chloropicrin and biosolarization with chicken manure.

Estimation of RKN soil densities present in the field before any nematicidal treatment (P0) is a valuable tool for decision-making in integrated nematode management since growers can decide nematicidal treatments to be used or crops and cultivar to be planted based on RKN soil population levels. A balance should be kept between the cost of nematicidal treatments and their efficacies in reducing RKN soil populations to maximize profitability in the field, even at high RKN soil infestations.

## Figures and Tables

**Table 1:** Nematicidal efficacies and costs of nematode management treatments.

Treatment	Efficacy (%)	Cost (€/ha)
1,3-Dichloropropene 81% + chloropicrin 44%	81.66 ± 1.58	1550
Dimethyl disulphide 95%	74.86 ± 1.64	N/A
Metam sodium 40%	51.23 ± 3.15	850
Abamectin 2%	42.60 ± 8.83	190
Azadirachtin 1%	54.60 ± 8.30	210
Azadirachtin 2.6%	54.72 ± 6.47	220
Dazomet 98%	51.16 ± 6.04	1980
Fenamiphos 24%	55.41 ± 3.29	970
Fluazaindolizine 50%	58.42 ± 4.99	N/A
Fluopyram 40%	63.70 ± 5.39	220
Fosthiazate 10%	51.14 ± 11.23	830
Fosthiazate 15%	57.25 ± 8.64	770
Oxamyl 10%	61.30 ± 4.25	160
Garlic extract 45%	40.08 ± 15.20	610
Garlic extract 100%	45.92 ± 10.51	190
Geraniol 12.1% + thymol 4.1%	48.27 ± 13.50	460
Biosolarization with chicken manure	72.13 ± 1.48	2700

Schneider–Orelli nematicidal corrected efficacies; values expressed as average ± standard error of 12 replicates. N/A: data not available.

**Table 2:** Costs, yields, revenues, and net returns for vegetable crops in intensive vegetable cultivation in southern Spain for the 2019, 2020, and 2021 seasons [12].

	Cost (€/ha)	Yield (kg/ha)	Revenue (€/ha)	Net Return (€/ha)
Aubergine	42,610	131,500	69,040	26,430
Cucumber	34,090	106,500	59,110	25,020
Melon	14,990	48,000	24,960	9970
Watermelon	17,000	70,000	27,650	10,650
Pepper	36,250	77,500	60,060	23,810
Tomato	51,600	113,500	78,320	26,720
Zucchini	22,910	70,000	37,100	14,200

**Table 3:** Average Seinhorst parameters for main vegetable–*M*. *incognita* pathosystems.

Crop	Tolerance Limit	Minimum Yield (%)	Net Return at Highest RKN Pi (€/ha)	References
Aubergine	5.4	5.0	−39,150	[19]
Cucumber	0.1	20.0	−22,270	[20]
Melon	5.6	28.5	−7860	[21,22,23]
Pepper	41.2	48.4	−7180	[24,25]
Tomato	91.9	31.3	−27,070	[26,27,28,29,30]
Watermelon	1.3	27.0	−9530	[31]
Zucchini	1.3	43.3	−6830	[22,32]

Tolerance limits expressed as J2/100 cm^3^ soil. Original data given as J2/g soil were recalculated to J2/100 cm^3^ soil assuming an average soil density of 1.3 g/cm^3^.

**Table 4:** Root-knot nematode (*M*. *incognita*) economic threshold (ET) and net return losses (NRL) at ET for various nematicidal treatments in intensive solanaceous cultivation.

Treatment	Aubergine		Pepper		Tomato	
	ET	NRL	ET	NRL	ET	NRL
1,3-Dichloropropene 81% + chloropicrin 44%	51	5.9	137	6.5	155	5.8
Metam sodium 40%	30	3.2	92	3.6	126	3.2
Abamectin 2%	11	0.7	52	0.8	99	0.7
Azadirachtin 1%	11	0.8	54	0.9	100	0.8
Azadirachtin 2.6%	12	0.8	54	0.9	101	0.8
Dazomet 98%	64	7.5	167	8.3	173	7.4
Fenamiphos 24%	34	3.7	100	4.1	131	3.6
Fluopyram 40%	12	0.8	54	0.9	101	0.8
Fosthiazate 10%	29	3.1	91	3.5	125	3.1
Fosthiazate 15%	28	2.9	87	3.2	123	2.9
Oxamyl 10%	10	0.6	51	0.7	98	0.6
Garlic extract 45%	23	2.3	78	2.6	116	2.3
Garlic extract 100%	11	0.7	52	0.8	99	0.7
Geraniol 12.1% + thymol 4.1%	19	1.7	68	1.9	110	1.7
Biosolarization with chicken manure	87	10.2	218	11.3	205	10.1

ET: Nematode economic damage threshold expressed as J2/100 cm^3^ soil.

**Table 5:** Root-knot nematode (*M*. *incognita*) economic threshold (ET) and net return losses (NRL) at ET for various nematicidal treatments in intensive cucurbitaceous cultivation.

Treatment	Cucumber		Melon		Watermelon		Zucchini	
	ET	NRL	ET	NRL	ET	NRL	ET	NRL
1,3-Dichlpr. 81% + chloropicrin 44%	58	6.2	181	15.6	160	14.6	154	10.9
Metam sodium 40%	31	3.4	96	8.5	84	8.0	81	6.0
Abamectin 2%	7	0.8	25	1.9	19	1.8	18	1.3
Azadirachtin 1%	8	0.9	27	2.1	21	2.0	20	1.5
Azadirachtin 2.6%	8	0.9	28	2.2	22	2.1	21	1.6
Dazomet 98%	74	7.9	238	19.9	211	18.6	203	14.0
Fenamiphos 24%	36	3.9	110	9.7	96	9.1	93	6.8
Fluopyram 40%	8	0.9	28	2.2	22	2.1	21	1.6
Fosthiazate 10%	30	3.3	94	8.3	82	7.8	79	5.9
Fosthiazate 15%	28	3.1	87	7.7	76	7.2	73	5.4
Oxamyl 10%	6	0.6	22	1.6	16	1.5	16	1.1
Garlic extract 45%	22	2.4	69	6.1	60	5.7	58	4.3
Garlic extract 100%	7	0.8	25	1.9	19	1.8	18	1.3
Geraniol 12.1% + thymol 4.1%	17	1.8	53	4.6	45	4.3	43	3.2
Biosolarization with chicken manure	103	10.8	346	27.1	306	25.4	293	19.0

ET: nematode economic damage threshold expressed as J2/100 cm^3^ soil.

**Table 6:** Maximum pre-treatment RKN (*M*. *incognita*) densities (P0) that each nematicidal treatment could manage to get RKN densities at planting (Pi) equal to the economic threshold level in intensive solanaceous crops in southern Spain.

Treatment	Aubergine	Pepper	Tomato
1,3-Dichlpr. 81% + chloropicrin 44%	398	1070	1211
Metam sodium 40%	61	188	257
Abamectin 2%	19	91	172
Azadirachtin 1%	24	119	220
Azadirachtin 2.6%	27	119	223
Dazomet 98%	154	401	415
Fluopyram 40%	33	149	278
Fenamiphos 24%	76	224	294
Fosthiazate 10%	59	186	256
Fosthiazate 15%	65	204	288
Oxamyl 10%	26	132	253
Garlic extract 45%	38	130	194
Garlic extract 100%	20	96	183
Geraniol 12.1% + thymol 4.1%	37	131	213
Biosolarization with chicken manure	322	807	759

RKN densities (P0) expressed as J2/100 cm^3^ soil.

**Table 7:** Maximum pre-treatment RKN (*M*. *incognita*) densities (P0) that each nematicidal treatment could manage to get RKN densities at planting (Pi) equal to the economic threshold level in intensive cucurbitaceous crops in southern Spain.

Treatment	Cucumber	Melon	Watermelon	Zucchini
1,3-Dichlpr. 81% + chloropicrin 44%	453	1414	1250	1203
Metam sodium 40%	63	196	171	165
Abamectin 2%	12	44	33	31
Azadirachtin 1%	18	59	46	44
Azadirachtin 2.6%	18	62	49	46
Dazomet 98%	178	572	507	488
Fenamiphos 24%	81	247	215	209
Fluopyram 40%	22	77	61	58
Fosthiazate 10%	61	192	168	162
Fosthiazate 15%	65	204	178	171
Oxamyl 10%	16	57	41	41
Garlic extract 45%	37	115	100	97
Garlic extract 100%	13	46	35	33
Geraniol 12.1% + thymol 4.1%	33	102	87	83
Biosolarization with chicken manure	381	1281	1133	1085

RKN densities (P0) expressed as J2/100 cm^3^ soil.

**Table 8:** Soil disinfestation treatments evaluated against *M*. *incognita* in field trials.

Treatment	Formulation	Dosage
Untreated control	-	-
1,3-Dichloropropene 81% + Chloropicrin 44%	Emulsifiable concentrate	300 kg/ha
Dimethyl Disulphide 95%	Emulsifiable concentrate	600 kg/ha
Metam Sodium 40%	Suspension concentrate	380 L/ha
Abamectin 2%	Suspension concentrate	5 L/ha
Azadirachtin 1%	Emulsifiable concentrate	3.9 L/ha
Azadirachtin 2.6%	Emulsifiable concentrate	1.5 L/ha
Dazomet 98%	Granules	350 kg/ha
Fenamiphos 24%	Capsule suspension	20 L/ha
Fluazaindolizine 50%	Suspension concentrate	1 kg/ha
Fluopyram 40%	Suspension concentrate	0.375 L/ha
Fosthiazate 10%	Granules	30 kg/ha
Fosthiazate 15%	Suspension concentrate	10 L/ha
Oxamyl 10%	Soluble concentrate	10 L/ha
Garlic extract 45%	Granules	25 kg/ha
Garlic extract 100%	Suspension concentrate	4 L/ha
Geraniol 12.1% + thymol 4.1%	Suspension concentrate	9 L/ha
Biosolarization with chicken manure	Organic amendment	20,000 kg/ha

## Data Availability

Not applicable.
